# Brucella suis vaccine strain 2 promotes intracellular proliferation by inducing M2 macrophage polarization and autophagy through PI3K/Akt/mTOR signalling

**DOI:** 10.1099/jmm.0.002182

**Published:** 2026-08-27

**Authors:** Haining Li, Juan Yang, Ting Xu, Zhang Ji, Zhenhai Wang

**Affiliations:** 1 Department of Neurology, The General Hospital of Ningxia Medical University, Yinchuan, Ningxia Hui Autonomous Region 750004, PR China; 2 Wuzhong City People’s Hospital, Wuzhong, Ningxia Hui Autonomous Region 750004, PR China; 3 Diagnosis and Treatment Engineering Technology Research Center of Nervous System Diseases of Ningxia, institute of Medical Scienes, General Hospital of Ningxia Medical University, Yinchuan 750004, PR China; 4 Neurology Center, General Hospital of Ningxia Medical University, Yinchuan, Ningxia, PR China

**Keywords:** autophagy, *Brucella suis* S2, BV2 cells, PI3K/Akt/mTOR, polarization

## Abstract

**Introduction.** The persistent intracellular survival of *Brucella* is a key factor contributing to immune evasion and chronic infection as evidenced by clinical recurrence. This ability of establishing long-term intracellular residency is a major driver of brucellosis relapse. Pathogens such as *Brucella* often manipulate host cell polarization and exploit autophagy to sustain their intracellular survival.

**Hypothesis/Gap Statement.** Real-world understanding of how macrophage polarization and autophagy jointly influence *Brucella* intracellular replication remains limited, and the specific signalling pathways mediating this interaction have not been fully characterized.

**Aim.** This study aimed to investigate the interplay between macrophage polarization, autophagy and intracellular proliferation of *Brucella suis* strain 2 and to characterize the involvement of the PI3K/AKT/mTOR signalling pathway in this process.

**Methodology.** We examined infection-induced alterations in BV2 cell polarization and autophagic activity following *Brucella* S2 infection. Autophagy-related protein expression (LC3B-I, LC3B-II, ULK1 and p62) was assessed by Western blot, and autophagosome-to-autolysosome conversion was visualized by laser confocal microscopy. Macrophage polarization markers (iNOS and Arg1) and cytokine secretion (TNF-*α* and IL-10) were measured by ELISA. Intracellular bacterial replication was quantified by c.f.u. assays, and pharmacological inhibitors were used to modulate PI3K/Akt/mTOR signalling and autophagic activity.

**Results.** Following *Brucella* S2 infection in BV2 cells, we observed elevated expression of iNOS, Arg1, p-Akt, mTOR and autophagy-related proteins (LC3B-I, LC3B-II and ULK1), accompanied by decreased p62 levels. Laser confocal microscopy revealed efficient autophagosome-to-autolysosome conversion. Time-dependent increases in TNF-*α* and IL-10 secretion indicated macrophage plasticity and the establishment of a mixed M1/M2 phenotype. c.f.u. assays demonstrated that PI3K/Akt/mTOR inhibition and autophagy induction promoted *Brucella* S2 intracellular proliferation. Notably, pharmacological inhibition of the PI3K/Akt/mTOR pathway reversed this effect by promoting M1 polarization while suppressing autophagic activity.

**Conclusion.** Collectively, our findings demonstrate that *Brucella* S2 infection induces both M1/M2 mixed polarization and autophagic activation in BV2 cells. Importantly, we identified that the M2-polarized state and autophagy induction synergistically promote intracellular bacterial replication, a process mediated through the PI3K/Akt/mTOR signalling pathway. These results suggest that targeted modulation of PI3K/Akt/mTOR signalling could represent a promising therapeutic approach to control *Brucella* S2 intracellular proliferation.

## Data Summary

The datasets used and/or analysed during the current study are available from the corresponding author on reasonable request.

## Introduction

*Brucella* spp. represent significant zoonotic pathogens with global distribution, including endemic regions in China [1–3]. Ningxia, as one of China’s brucellosis epidemic areas, reports numerous clinical cases characterized by diverse manifestations and frequent recurrence – a phenomenon potentially attributable to the bacterium’s intracellular persistence [4]. Despite its clinical importance, brucellosis remains understudied, particularly regarding mechanisms facilitating *Brucella*’s sustained intracellular survival [5, 6]. This facultative intracellular pathogen demonstrates remarkable adaptability, capable of survival and proliferation in both phagocytic and non-phagocytic cells [7, 8]. During early infection of macrophages and epithelial cells, *Brucella* establishes specialized membrane-bound compartments [*Brucella*-containing vacuoles (BCVs)] that undergo maturation into replicative BCVs (rBCVs) [9–11]. These ER-derived replicative niches strategically evade lysosomal fusion, enabling bacterial persistence [12]. Macrophage activation constitutes the primary immune defence during initial pathogen encounter [13, 14]. However, *Brucella* subverts this critical host response, manipulating macrophage polarization to create a permissive intracellular environment essential for its survival and propagation.

Macrophages exhibit remarkable plasticity, differentiating into distinct functional phenotypes. The two best-characterized subsets are classically activated M1 macrophages (exhibiting potent microbicidal activity) and alternatively activated M2 macrophages (possessing immunoregulatory functions) [15, 16]. As tissue-resident phagocytes of the central nervous system, microglia share this polarization capacity. Given our focus on neurobrucellosis, we selected the BV2 microglial cell line as our experimental model. M1 polarization is marked by inducible nitric oxide synthase (iNOS) expression and proinflammatory cytokine production, while M2 macrophages predominantly express arginase-1 (Arg1) [17]. The dynamic equilibrium between M1/M2 populations is crucial for effective pathogen clearance while minimizing tissue damage. Disruption of this balance often underlies infection-associated pathology. Bacterial pathogens modulate host immunity by altering inflammatory cytokine networks, with disease outcomes largely determined by the interplay between pro- and anti-inflammatory mediators. Temporal regulation of these cytokines is particularly critical during early infection, influencing both bacterial clearance and host resistance. Our previous work demonstrated that *Brucella* outer membrane protein 31 (OMP31) triggers inflammatory responses in BV2 cells [18]. Building on these findings, the current study investigates *Brucella*-induced polarization shifts in BV2 cells and their functional consequences for intracellular bacterial proliferation.

Emerging evidence indicates that autophagy-related proteins play crucial roles in the biogenesis of rBCVs and facilitate the completion of the intracellular life cycle [19, 20]. Interestingly, rBCV formation depends on autophagosome initiation components (Beclin1, PI3K and ULK1) but appears independent of elongation machinery (Atg5, Atg7, LC3B and Atg4B), suggesting involvement of alternative autophagic pathways. While the precise mechanisms remain unclear, studies demonstrate that ULK1 and Beclin1 participate in noncanonical autophagy pathways that do not require LC3, Atg7 or Atg5 recruitment. Although autophagy typically functions as an antimicrobial defence, numerous intracellular pathogens have evolved strategies to co-opt autophagic processes for their survival. Similar to other intracellular bacteria, *Brucella* establishes replication-permissive compartments, yet the nature of its interaction with host autophagy remains controversial, particularly regarding autophagy’s impact on bacterial replication [21]. Elucidating how *Brucella* modulates autophagy could yield critical insights for developing novel therapeutic strategies against brucellosis [22, 23]. Building on our previous finding that *Brucella* OMP31 induces autophagosome formation in BV2 cells, the current study specifically investigates autophagy dynamics during *Brucella suis* strain 2 (*B. suis* S2) infection. Using BV2 microglial cells as a model system, we systematically examined (1) autophagy flux alterations following S2 infection, (2) the relationship between autophagic activity and intracellular bacterial replication and (3) the underlying molecular mechanisms governing these processes.

## Methods

### Cell line and culture conditions

The murine microglial BV2 cell line was maintained in Dulbecco’s modified Eagle’s medium (Gibco, Carlsbad, CA, USA) supplemented with 10% heat-inactivated foetal bovine serum (HyClone, Logan, UT, USA), 2 mM l-glutamine and 200 U ml^−1^ penicillin–streptomycin (complete growth medium). Cells were cultured at 37 °C in a humidified atmosphere containing 5% CO2.

### Bacterial strain and culture conditions

*B. suis* S2 was kindly provided by Professor Xu (Ningxia Medical University, Yinchuan, China). The lyophilized bacterial stock was reconstituted in sterile tryptic soy broth (TSB) and plated on tryptic soy agar (TSA) medium, followed by incubation at 37 °C with 5% CO₂. For experimental use, a single colony was inoculated into sterile TSB and cultured under the same conditions for approximately 24 h. Bacterial cells were harvested by centrifugation (2,000 ***g***, 20 min, room temperature) and washed twice with PBS. Bacterial concentrations were determined using McFarland standards (bioMérieux, Beijing, China). All procedures were performed in a Biosafety Level 2 facility.

### *In vitro* infection protocol

BV2 cells were plated in 6-well culture plates at a density of 1×10⁴ cells per well. When cells reached ~50% confluency, they were infected with *B. suis* S2 at a multiplicity of infection (MOI) of 200 in antibiotic-free medium for 1 h. Following infection, cells were thoroughly washed with PBS to remove non-internalized bacteria. The infected cultures were then maintained in complete medium containing 100 µg ml^−1^ gentamicin for various time points to eliminate any remaining extracellular bacteria while allowing intracellular bacterial proliferation.

### Western blot analysis

Following PBS washing (twice), cellular proteins were extracted using RIPA lysis buffer. Protein quantification was performed using the bicinchoninic acid assay (Keygene, Nanjing, China) following the manufacturer’s protocol. Equal amounts of protein (30 µg per lane) were resolved by 10% SDS-PAGE (Mini-Protean TGX gels, Bio-Rad) and electrophoretically transferred to polyvinylidene difluoride membranes using standard transfer conditions. Membranes were probed overnight at 4 °C with the following primary antibodies (all from Abcam, Cambridge, MA): anti-Beclin1 (1 : 1,000), anti-LC3B (1 : 1,000; detects both LC3B-I and LC3B-II isoforms), anti-p62 (1 : 1,000), anti-ULK1 (1 : 1000), anti-mTOR (1 : 1,000) and anti-GAPDH (1 : 5,000; loading control). After washing, membranes were incubated with Horseradish Peroxidase (HRP)-conjugated goat anti-mouse secondary antibodies (1 : 5,000; Zhongshan Golden Bridge, Beijing, China) for 1 h at room temperature. Protein bands were visualized using enhanced chemiluminescence (Western Lightning Ultra, Bio-Rad) and imaged using the EpiChemi3 Darkroom System (UVP BioImaging Systems). Densitometric analysis was performed using ImageJ software (NIH, USA).

### Reverse transcription-quantitative PCR

Cellular RNA was extracted with an RNA extraction kit (Tiangen Biotech, China). A RevertAid First Strand cDNA Synthesis Kit (Thermo Fisher Scientific Inc.) was used to reverse-transcribe total RNA into cDNA. mRNA expression was quantified using a First Strand cDNA synthesis kit (Thermo Fisher Scientific Inc.). The expression of LC3B and Beclin1 was determined using a PrimeScript RT-PCR kit (DBI Bioscience). GAPDH was used as the internal reference. All primers were purchased from Sangon Biotech (Shanghai, China) and are shown in Table 1. Three replicates were used for each sample.

**Table 1.** Primer sequences for RT‐qPCR

Target gene	Primer sequence (5′-3′)
LC3B	F:-TTCTTCCTCCTGGTGAATGG-
LC3B	R:-ATTGCTGTCCCGAATGTCTC-
Beclin1	F:-AGCCTCTGAAACTGGACACG-
Beclin1	R:CCTCTTCCTCCTGGGTCTCT
iNOS	F:-AGCCTCTGAAACTGGACACG-
iNOS	R:-GAGCACGCTGAGTACCTCATTG-
Arg1	F:-AGCATCCACCCAAATGACAC-
Arg1	R:-GCAGAGGTCCAGAAGAATGG-
GAPDH	F:CAAGGTCATCCATGACAACTTTG
GAPDH	R:GTCCACCACCCTGTTGCTGTAG

### Cell viability assay

Cell viability was assessed using the Cell Counting Kit-8 (CCK-8) (BestBio, Shanghai, China) following the manufacturer’s instructions. Briefly, cells (5×10³ per well) were seeded in 96-well plates and allowed to adhere overnight. Subsequently, the cells were treated with varying concentrations of Rapa (1, 10, 100 and 1,000 nM) and 3-MA (1, 2, 4 and 8 mM) for 36 h. After treatment, CCK-8 solution was added to each well, and the plates were incubated for an additional 4 h. Absorbance was then measured at 450 nm using a microplate reader (BioTek, Winooski, VT, USA).

### Laser scanning confocal microscopy

Confocal imaging was performed using an Olympus laser scanning microscope. BV2 cells were seeded in a 6-well plate and divided into two groups: a control group and an S2-infected group. Following adherence, the cells underwent standard procedures for climbing, fixation and coverslip mounting. Green (GFP) and red fluorescent protein (RFP) signals were excited at 561 nm. Image acquisition and processing were conducted using the FV10-ASW 1.7 Viewer software.

### Bacterial c.f.u. enumeration assay

TSA was prepared following the manufacturer’s specifications and sterilized by autoclaving at 121 °C for 30 min. Under sterile laminar flow conditions, ~25 ml of molten TSA (cooled to 50–55 °C) was aseptically dispensed into 90-mm diameter sterile Petri dishes. The plates were allowed to solidify at room temperature, then inverted and stored at 4 °C until use. For bacterial quantification, BV2 microglial cells were infected with *B. suis* S2 at an MOI of 200 for the specified duration. Post-infection, cells were washed twice with PBS (pH 7.4) and lysed with 0.3% (v/v) Triton X-100 in PBS for 10 min at room temperature to release intracellular bacteria. The resulting lysates were serially diluted (tenfold dilutions in PBS), and 100 µl aliquots were spread-plated onto TSA plates in triplicate. Plates were incubated aerobically at 37 °C with 5% CO₂ for 96 h. Following incubation, distinct colonies were manually enumerated, and c.f.u. per millilitre values were calculated from countable plates (containing 30–300 colonies per plate). Statistical analysis was performed using GraphPad Prism version X.X (GraphPad Software, San Diego, CA), with data presented as mean±sd from at least three independent experiments.

### ELISA analysis

Following infection with *Brucella suis* strain S2 (S2) and treatment with rapamycin (Rapa), 3-methyladenine (3-MA) or LY294002 (LY) as indicated, cell culture supernatants were collected by centrifugation at 300 ***g*** for 10 min at 4 °C. The concentrations of pro-inflammatory cytokines (IL-1*β*, IL-12 and TNF-*α*) and anti-inflammatory cytokine IL-10 were quantified using commercial ELISA kits (Neobioscience Technology, Shenzhen, China) according to the manufacturer’s instructions. Briefly, 100 µl of supernatant or standard was added to antibody-coated wells and incubated for 2 h at 37 °C. After washing, biotinylated detection antibody was added, followed by streptavidin-HRP conjugate. Tetramethylbenzidine substrate was used for colour development, and the reaction was stopped with 2 N H₂SO₄. Absorbance was measured at 450 nm with a reference wavelength of 570 nm using a microplate reader (BioTek Instruments). All samples were assayed in triplicate, and cytokine concentrations were determined from standard curves generated for each plate. Data represent the mean±sd of three independent experiments, each performed with technical triplicates.

### Statistical analysis

All quantitative data are expressed as mean±sd derived from a minimum of three biologically independent experiments. Statistical analyses were performed using GraphPad Prism software (version 9.5.0; GraphPad Software, San Diego, CA, USA). For multiple group comparisons, ANOVA with Tukey’s honest significant difference post hoc test was applied. A two-tailed probability value of *P*<0.05 was considered statistically significant. All statistical tests were conducted assuming normal distribution and homogeneity of variance, with appropriate verification of these assumptions.

## Results

### *B. suis* S2 infection induces PI3K/Akt/mTOR activation, driving BV2 cells towards an M1/M2 phenotype

**Fig. 1.**
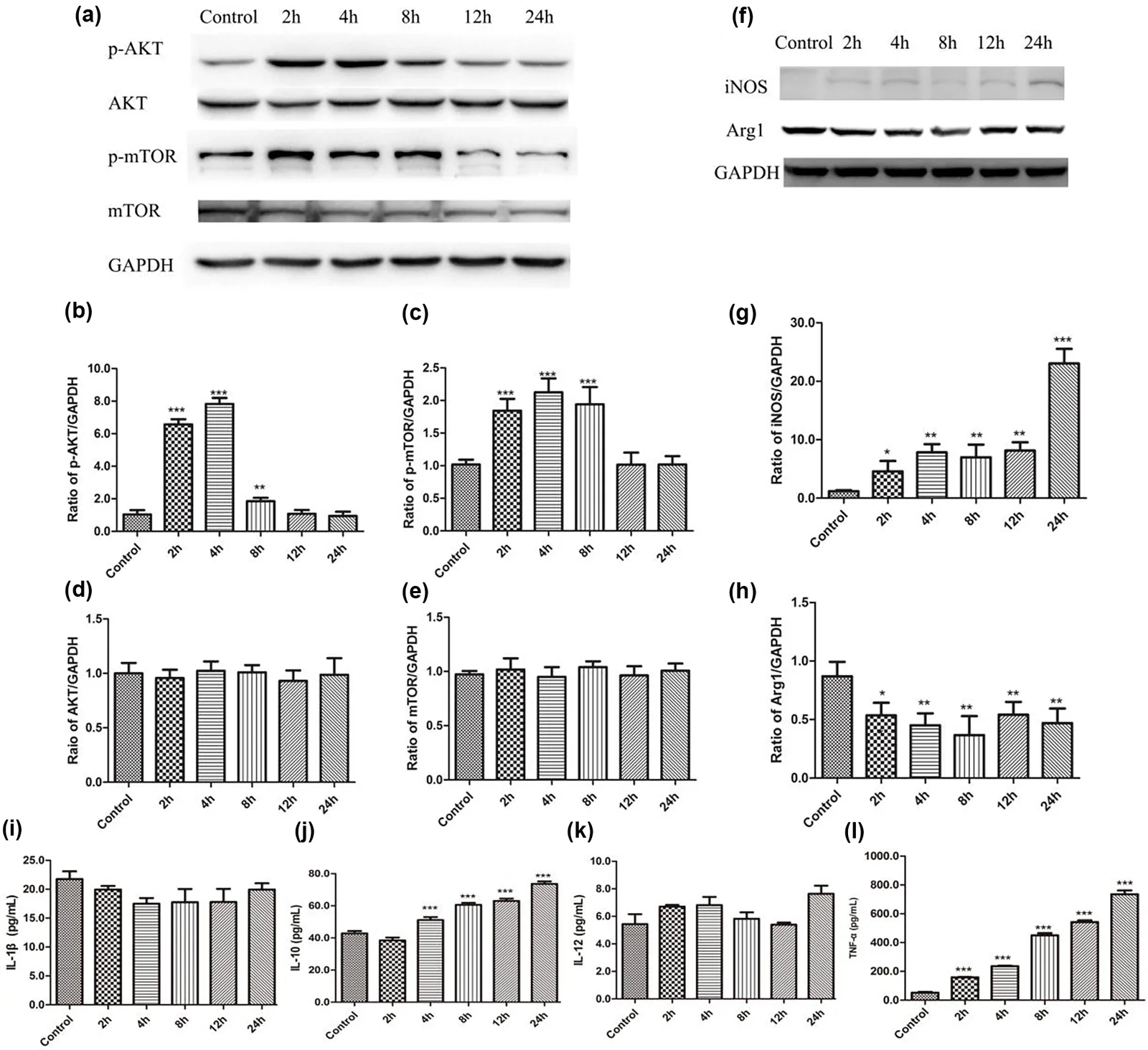
*B. suis* S2 infection activates the PI3K/Akt/mTOR pathway and induces M1/M2 mixed polarization in BV2 microglial cells. (**P<0.01, ***P<0.001 vs. control group). (**a, f**) Representative immunoblots of Akt, mTOR, iNOS and Arg1 expression. (**b, c, d, e, g, h**) Densitometric quantification of Akt, mTOR, iNOS and Arg1 protein levels normalized to *β*-actin (mean±sd, *n*=3). (**i, j, k, l**) ELISA analysis of IL-1*β*, TNF-*α*, IL-12 and IL-10 secretion at different time points post-infection.

A BV2 cell model of *B. suis* S2 infection was established as previously described [24]. We first examined the temporal activation of the PI3K/Akt/mTOR signalling pathway within 24 h post-infection (hpi). Western blot analysis revealed that PI3K/Akt/mTOR signalling was activated in a time-dependent manner, with phospho-Akt (p-Akt) peaking at 4 hpi before gradually declining to baseline levels by 12 hpi (Fig. 1a, b). Similarly, phospho-mTOR expression peaked at 4 hpi and returned to control levels by 12 hpi (Fig. 1a, c).

To assess BV2 cell polarization, we measured the expression of M1 markers (iNOS, TNF-*α*, IL-1*β* and IL-12) and M2 markers (Arg1 and IL-10). Western blotting showed that iNOS (M1-associated) increased significantly by 2 hpi and remained elevated up to 24 hpi (Fig. 1f, g), whereas Arg1 (M2-associated) increased at 4 hpi and persisted until 24 hpi (Fig. 1f, h), with both showing significant differences compared to controls. ELISA results further demonstrated that TNF-*α* (pro-inflammatory) levels rose sharply at 2 hpi and continued increasing over time (*P*<0.001) (Fig. 1l). IL-1*β* and IL-12 (M1-related cytokines) showed no significant changes (Fig. 1i, k). IL-10 (anti-inflammatory) increased from 4 hpi in a time-dependent manner (Fig. 1j). Collectively, these findings indicate that *B. suis* S2 infection activates the PI3K/Akt/mTOR pathway and drives BV2 microglia towards a mixed M1/M2 phenotype.

### Induction of autophagy following *B. suis* S2 infection

**Fig. 2.**
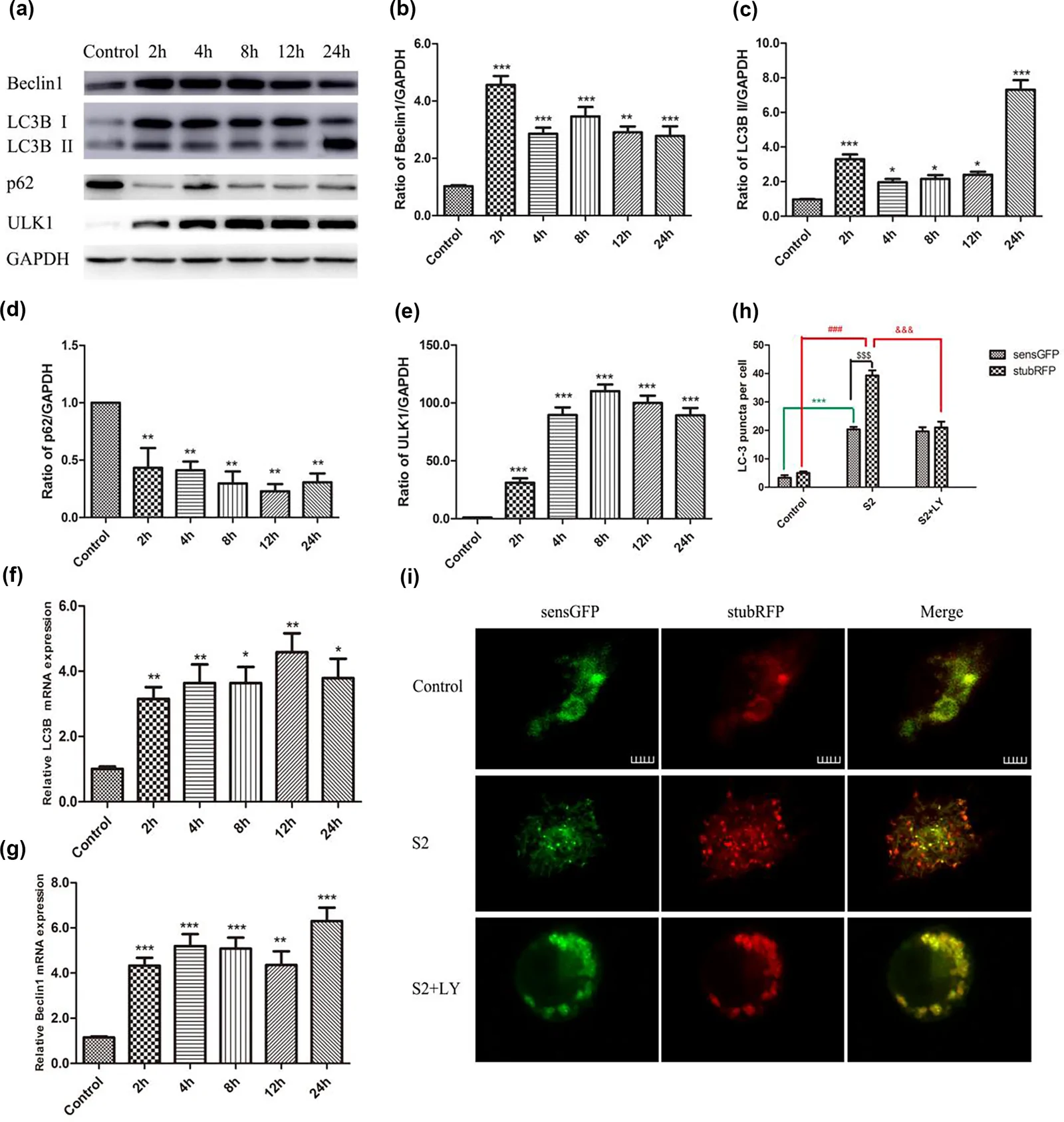
Autophagy induction in BV2 microglial cells following *B. suis* S2 infection. (*P<0.05, **P<0.01, ***P<0.001 vs. control group). Representative Western blots of Beclin1, LC3B, p62 and ULK1 protein expression (**a–e**), Densitometric quantification of protein levels normalized to *β*-actin (mean±sd, *n*=3). (**f, g**) Reverse transcription-qPCR analysis of LC3B and Beclin1 mRNA expression with GAPDH as internal control (mean±sd, *n*=9; *P<0.05, **P<0.01, *P<0.001 vs. control). (**h, i**) Confocal microscopy images showing StubRFP-SensGFP-LC3 distribution (*P<0.001, ###*P*<0.001 vs. control; $$$*P*<0.001, SensGFP vs. StubRFP in S2 group). The S2+LY group results will be presented in the following section.

To comprehensively assess autophagy induction, we examined autophagic protein expression, autophagic flux and autophagosome formation in BV2 cells at 2, 4, 8, 12 and 24 hpi with *B. suis* S2. Western blot analysis revealed that Beclin1, LC3B and ULK1 – key markers of autophagy were significantly upregulated within 24 hpi compared to the control group (Fig. 2a–c and e). Conversely, p62, a protein degraded during autophagy, was markedly reduced (Fig. 2a, d). Consistent with these findings, quantitative PCR (qPCR) analysis demonstrated that mRNA levels of Beclin1 and LC3B followed similar trends (Fig. 2f, g), further supporting autophagy induction.

To evaluate autophagic flux, we employed confocal laser microscopy to monitor LC3 (green fluorescence and autophagosomes) and lysosomal (red fluorescence) signals. Under basal conditions, few fluorescent puncta were observed. However, post-infection, both LC3-positive autophagosomes (green, *P*<0.001) and lysosomal signals (red, *P*<0.001) increased significantly. Notably, green puncta were fewer than red puncta (*P*<0.001), indicating efficient autophagosome-lysosome fusion and enhanced autophagic flux (Fig. 2h, i). Together, these data demonstrate that *B. suis* S2 infection robustly induces autophagy in BV2 cells, as evidenced by increased autophagic protein expression, elevated LC3 lipidation, p62 degradation and enhanced autophagic flux.

### PI3K inhibition promotes M2 polarization and suppresses autophagy in BV2 cells

To investigate the role of PI3K signalling in microglial polarization and autophagy, we treated BV2 cells with the PI3K inhibitor LY prior to *B. suis* S2 infection. We examined alterations in microglial polarization-associated proteins while simultaneously evaluating cytokine profiles and autophagy modulation.

**Fig. 3.**
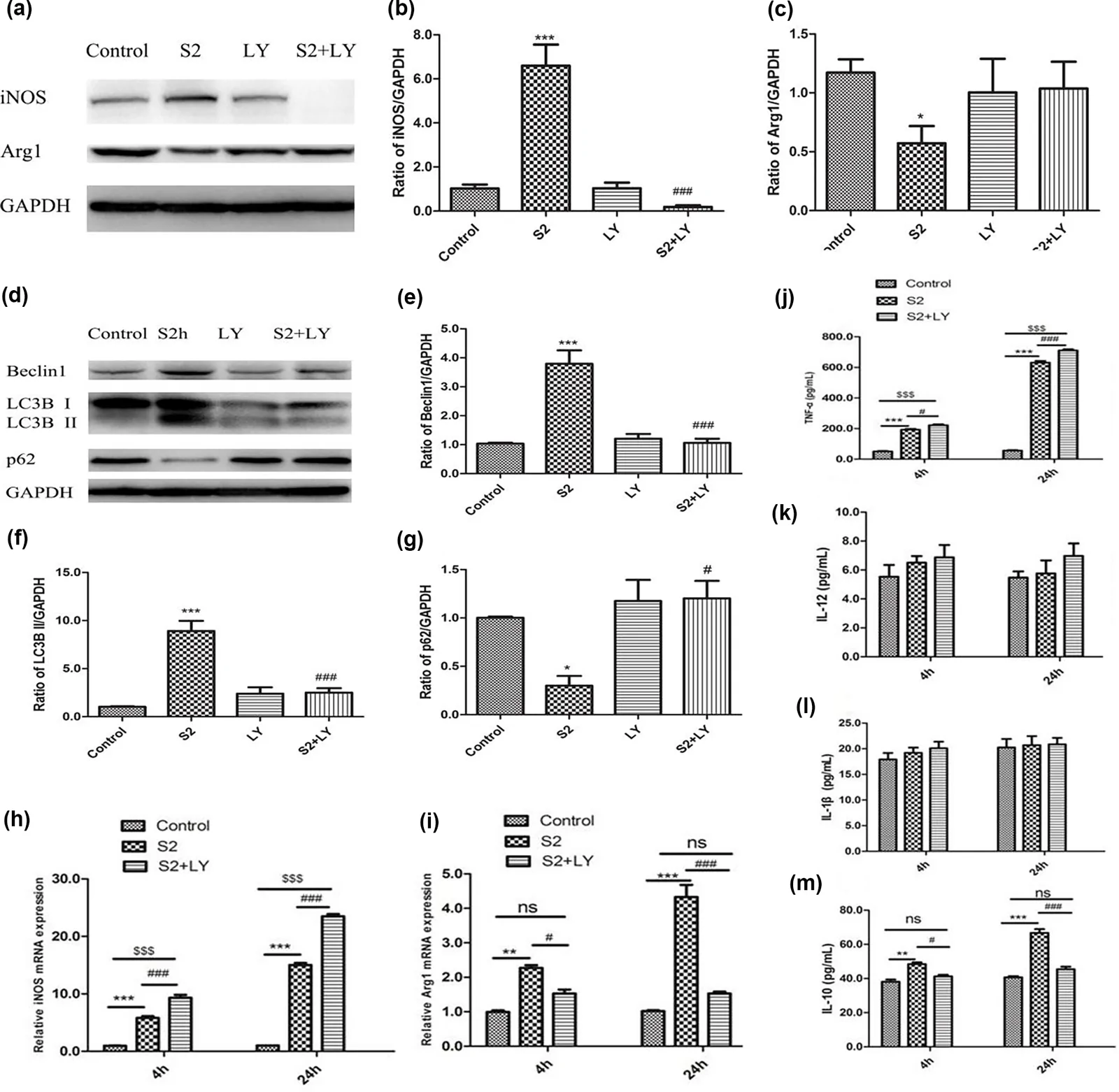
Effects of LY treatment on polarity and autophagy in *B. suis* S2-infected BV2 microglial cells. (a–c) Western blot analysis of iNOS and Arg1 protein expression in BV2 microglia. Densitometric data normalized to *β*-actin are presented as mean±sd (*n*=3). *P<0.01, ***P<0.001 vs. control group; ###*P*<0.001 vs. S2 group. (d–g) Inhibitory effects of *B. suis* S2 on Beclin1, LC3B and p62 protein levels in BV2 microglial cells. *P<0.05, ***P<0.001 vs. control group; #*P*<0.05, ###*P*<0.001 vs. S2 group. (**h, i**) Reverse transcription-qPCR analysis of iNOS and Arg1 mRNA levels in BV2 microglia, with GAPDH as an internal control (mean±sd, *n*=9). (j–m) ELISA quantification of TNF-*α*, IL-1*β*, IL-12 and IL-10 levels. *P<0.05, **P<0.01, ***P<0.001 vs. control group; $$$*P*<0.001 (control vs. S2+LY group); #*P*<0.05, ###*P*<0.001 (S2+LY vs. S2 group); **P<0.01, ***P<0.001 (control vs. S2 group).

Western blot analysis revealed that S2 infection significantly increased iNOS protein expression (M1 marker), which was markedly attenuated by LY pretreatment (*P*<0.001 vs. S2 group, Fig. 3a, b). Conversely, Arg1 protein levels (M2 marker) decreased following LY treatment, showing no significant difference from control levels (Fig. 3a, c). Interestingly, while iNOS mRNA expression was higher in LY-treated cells than in the S2 infection group (Fig. 3h), Arg1 mRNA levels were significantly lower than in infected cells (Fig. 3i), suggesting post-transcriptional regulation. LY treatment enhanced TNF-*α* production compared to S2 infection alone (*P*<0.05, Fig. 3j), but did not affect IL-1*β* (Fig. 3l) or IL-12 (Fig. 3k) expression. Notably, LY reduced IL-10 levels (Fig. 3m), indicating a complex effect on inflammatory responses. LY pretreatment significantly reversed S2-induced autophagy markers: Beclin1 upregulation at 4 hpi was attenuated (*P*<0.001 vs. S2 group, Fig. 3d, e). LC3B-II accumulation was reduced (*P*<0.05; Fig. 3d, f). p62 degradation was inhibited (*P*<0.05, Fig. 3d, g).

### Inhibition of PI3K/Akt/mTOR signalling suppresses *B. suis* S2 replication in murine microglial BV2 cells

**Fig. 4.**
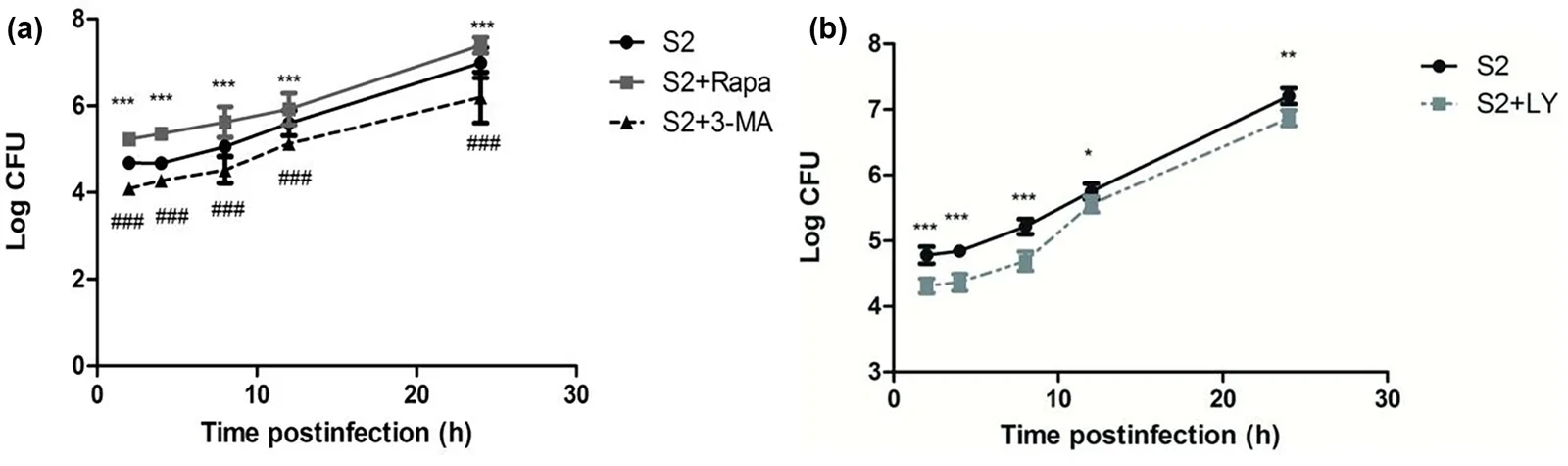
Effects of Rapa, 3-MA and LY treatment on intracellular replication of *B. suis* S2 in microglial cells.(**a**) ***P<0.001 for S2+Rapa group vs. S2 group; ###*P*<0.001 for S2+3 MA group vs. S2 group. (**b**) *P<0.05, ***P<0.001 vs. S2 group.

To investigate the effect of PI3K/mTOR pathway inhibition on *Brucella* S2 proliferation, BV2 cells were pretreated for 1 h with LY (PI3K inhibitor), Rapa (mTOR inhibitor) or 3-MA (autophagy inhibitor), followed by infection with *Brucella* S2. Intracellular bacterial loads were quantified at 2, 4, 8, 12 and 24 hpi using c.f.u. assays. As shown in Fig. 4a, b, both the S2+LY and S2+3 MA treatment groups exhibited significantly lower logCFU values compared to the S2-only group at all time points (*P*<0.05). These results demonstrate that pharmacological inhibition of PI3K (via LY) or autophagy (via 3-MA) effectively restricts *Brucella* S2 replication within BV2 microglial cells.

### M2 phenotype polarization promotes intracellular proliferation of *B. suis* S2

**Fig. 5.**
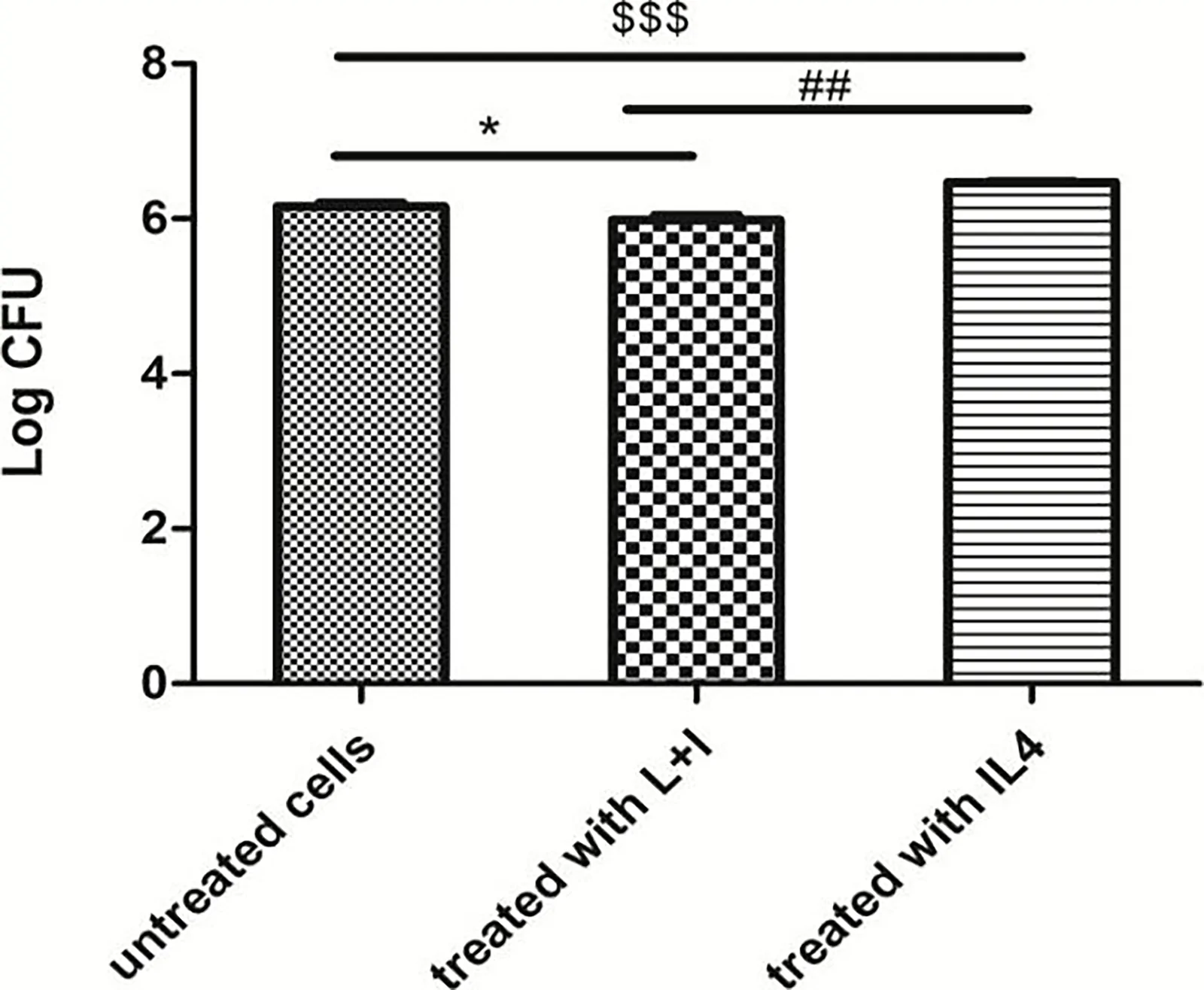
Intracellular growth capacity of *B. suis* S2 in microglial cells under different polarization conditions. L+I means LPS+IFN-*γ*. *P<0.001, vs. untreated group, $$$*P*<0.001, vs. untreated group, ##*P*<0.05, treated with L+I group vs. treated with IL4 group.

Our findings demonstrate that *B. suis* S2 infection induces both M1 and M2 polarization in BV2 microglial cells. To further investigate the relationship between macrophage polarization and bacterial proliferation, we treated BV2 cells with specific polarizing agents: LPS (100 ng ml^−1^) + IFN-*γ* (20 ng ml^−1^) for M1 polarization and IL-4 (20 ng ml^−1^) for M2 polarization. IL-4-induced M2 polarization significantly enhanced intracellular S2 proliferation, as evidenced by increased colony counts compared to the control group (*P*<0.001). Conversely, LPS+IFN-*γ*-induced M1 polarization markedly reduced bacterial loads (*P*<0.001, Fig. 5). These results strongly suggest that macrophage polarization state directly influences intracellular bacterial replication efficiency.

## Discussion

Research on *Brucella*-induced macrophage/microglia polarization remains limited. Our study demonstrates that *B. suis* S2 infection induces a mixed M1/M2 state in BV2 microglial cells. Following PI3K inhibition, we observed upregulation of M1 markers (iNOS gene expression), increased proinflammatory cytokine TNF-*α*, downregulation of M2 markers (Arg1 expression) and reduced anti-inflammatory cytokine IL-10. These findings suggest PI3K signalling promotes M1 polarization in BV2 cells. This aligns with Wang *et al*.’s work showing *Brucella* infection dysregulates macrophage polarization through LC3-dependent autophagy, impairing monocyte-derived macrophage function [23]. Parallels exist with other intracellular pathogens like *Mycobacterium tuberculosis*. Several studies indicate intracellular bacteria exploit macrophage plasticity to enhance their survival [25–27]. Notably, Andrade *et al*. demonstrated that virulent *Mycobacterium bovis* strains inhibit classical macrophage activation while inducing an M1/M2 hybrid phenotype through IFN-*γ*-mediated mechanisms. This phenotypic shift, characterized by increased Arg1 expression and reduced nitric oxide production, appears to protect bacteria from nitrosative stress [24]. Microglia exhibit remarkable plasticity, often displaying simultaneous expression of both M1 and M2 markers [17, 28, 29]. Our results underscore the complexity of microglial polarization, which cannot be adequately characterized using single-phenotype classifications. This challenges traditional binary views of macrophage polarization and highlights the need for more nuanced approaches to studying host–pathogen interactions in neurobrucellosis. It should be noted that our assessment of BV2 cell polarization is based on population-level assays. The observed mixed features may reflect either co-activation of M1 and M2 pathways within the same cells or a heterogeneous population with distinct M1 and M2 subsets. Our data cannot distinguish between these possibilities. Therefore, we use the descriptive term ‘a mixed activation state displaying both M1- and M2-associated features’ without equating it to classical polarization subtypes. Future single-cell analyses (e.g. scRNA-seq or phospho-flow) are needed to resolve microglial heterogeneity during *Brucella* infection.

The most evident indicator of macrophage polarization shifts is the alteration in cytokine profiles. As key mediators of innate and adaptive immune responses, cytokines play a pivotal role in host defence against *Brucella* infection [30, 31]. While the proinflammatory response and its underlying mechanisms have been extensively studied in *Brucella* infections – with most research focusing on immunomodulatory agents that promote M1 polarization – the impact of polarization shifts on bacterial proliferation remains unclear. Our findings demonstrate that inducing a switch to the M2 phenotype in BV2 cells facilitates the intracellular proliferation of *Brucella* S2, a process mediated by the PI3K/Akt/mTOR pathway. The upregulation of Arg1 and IL-10 reflects a protective mechanism by which host cells mitigate excessive inflammation. The PI3K/Akt/mTOR signalling pathway integrates diverse extracellular and intracellular signals to modulate macrophage biology, influencing pro- and anti-inflammatory cytokine production, phagocytosis and autophagy [26]. These processes collectively determine macrophage functional polarization, broadly classified into M1 and M2 phenotypes. In this study, inhibition of the PI3K/Akt/mTOR pathway led to a marked reduction in inflammatory factor secretion by BV2 cells, concomitant with decreased intracellular proliferation of *Brucella* S2. Furthermore, we observed a reciprocal regulatory relationship between PI3K/Akt/mTOR signalling and autophagy. Based on these findings, we propose that PI3K/Akt/mTOR contributes to autophagy modulation and governs BV2 cell polarization, ultimately influencing the intracellular replication of *Brucella* S2.

Autophagy serves as a critical component of the innate immune defence against intracellular pathogens such as *Brucella* [22, 25, 32]. However, its functional role varies depending on the bacterial species involved. For instance, *M. tuberculosis* employs multiple strategies to evade autophagic clearance, facilitating persistent infection and pathogenesis [21]. Emerging evidence suggests that the noncanonical autophagy pathway significantly influences host–pathogen interactions and shares upstream regulators with canonical autophagy [33, 34]. In our study, infection with *Brucella* S2 upregulated the autophagy marker proteins Beclin1 and LC3-II while downregulating p62 in BV2 microglia. This autophagic response correlated with intracellular bacterial proliferation. The conversion of LC3-I to LC3-II, a well-established indicator of autophagic activity, was confirmed by Western blot. Furthermore, confocal laser microscopy revealed increased autophagosome formation (GFP-RFP-LC3 puncta), indicating enhanced autophagic initiation rather than complete flux. Our c.f.u. data show that pharmacological or genetic inhibition of autophagy significantly reduces *Brucella* S2 survival, indicating that autophagy overall promotes bacterial replication. However, the canonical view considers autophagy a host defence mechanism. How to resolve this paradox? According to Starr *et al*. [35], *Brucella* employs a selective subversion strategy: it utilizes the autophagic initiation complex (ULK1-Beclin1-ATG14L-VPS34) to generate autophagic *Brucella*-containing vacuole (aBCV) replicative niches, while avoiding elongation/maturation machinery (e.g. ATG5, ATG7 and LC3B) to prevent lysosomal degradation. Thus, in our model, autophagic initiation is exploited to benefit *Brucella*, whereas complete flux (degradation) is blocked. This explains why inhibiting initiation (e.g. 3-MA or Beclin1 siRNA) reduces bacterial burden – bacteria cannot form protective aBCVs. In summary, *Brucella* subverts autophagy from a host defence into its own survival machine. Our net pro-bacterial effect is consistent with this model.

In summary, this study demonstrates that *Brucella* S2 infection triggers M1/M2 polarization and autophagy activation in BV2 cells, with different effects on bacterial intracellular proliferation. While the shift towards an M2 phenotype promotes *Brucella* survival, autophagy induction restricts bacterial replication – a process modulated by the PI3K/Akt/mTOR pathway. These findings not only elucidate the dual role of macrophage polarization and autophagy in *Brucella* infection but also propose a potential therapeutic strategy: targeting the PI3K/Akt/mTOR axis to enhance autophagic clearance and counteract M2-mediated immune evasion. By simultaneously regulating macrophage polarization and autophagy, this approach could mitigate intracellular bacterial proliferation, offering new insights for combating *Brucella* infections.

## Abbreviations

aBCV: autophagic *Brucella*-containing vacuole
Arg1: arginase-1
BCV: *Brucella*-containing vacuole
*B. suis* S2: *Brucella suis* strain 2
CCK-8: Cell Counting Kit-8
GFP: green fluorescent protein
hpi: h post-infection
iNOS: inducible nitric oxide synthase
LY: LY294002
3-MA: 3-methyladenine
MOI: multiplicity of infection
OMP31: outer membrane protein 31
p-Akt: phospho-Akt
qPCR: quantitative PCR
Rapa: rapamycin
rBCV: replicative *Brucella*-containing vacuoles
RFP: red fluorescent protein
TSA: tryptic soy agar
TSB: sterile tryptic soy broth
